# Secondary Mapping of Lymphatic Filariasis in Haiti-Definition of Transmission Foci in Low-Prevalence Settings

**DOI:** 10.1371/journal.pntd.0001807

**Published:** 2012-10-11

**Authors:** Naomi Drexler, Charles H. Washington, Maribeth Lovegrove, Caroline Grady, Marie Denise Milord, Thomas Streit, Patrick Lammie

**Affiliations:** 1 Rollins School of Public Health, Emory University, Atlanta, Georgia, United States of America; 2 Centers for Disease Control and Prevention, Division of Parasitic Diseases and Malaria, Atlanta, Georgia, United States of America; 3 Center for Tropical Research Disease and Training, University of Notre Dame, Notre Dame, Indiana, United States of America; 4 Ministry of Health and Population, Port au Prince, Haiti; University of Pittsburgh, United States of America

## Abstract

To eliminate Lymphatic filariasis (LF) as a public health problem, the World Health Organization (WHO) recommends that any area with infection prevalence greater than or equal to 1% (denoted by presence of microfilaremia or antigenemia) should receive mass drug administration (MDA) of antifilarial drugs for at least five consecutive rounds. Areas of low-antigen prevalence (<1%) are thought to pose little risk for continued transmission of LF. Five low-antigen prevalence communes in Haiti, characterized as part of a national survey, were further assessed for transmission in this study. An initial evaluation of schoolchildren was performed in each commune to identify antigen-positive children who served as index cases for subsequent community surveys conducted among households neighboring the index cases. Global positioning system (GPS) coordinates and immunochromatographic tests (ICT) for filarial antigenemia were collected on approximately 1,600 persons of all ages in the five communes. The relationship between antigen-positive cases in the community and distance from index cases was evaluated using multivariate regression techniques and analyses of spatial clustering. Community surveys demonstrated higher antigen prevalence in three of the five communes than was observed in the original mapping survey; autochthonous cases were found in the same three communes. Regression techniques identified a significantly increased likelihood of being antigen-positive when living within 20 meters of index cases when controlling for age, gender, and commune. Spatial clustering of antigen-positive cases was observed in some, but not all communes. Our results suggest that localized transmission was present even in low-prevalence settings and suggest that better surveillance methods may be needed to detect microfoci of LF transmission.

## Author Summary

Lymphatic filariasis (LF) is among the leading causes of disability among tropical diseases and is caused by a mosquito-transmitted parasite but can be prevented using mass drug therapy and vector-control. In recent years, an international effort has been mounted to eliminate LF. In order to focus limited resources on areas with the highest disease burden, the World Health Organization (WHO) has suggested that mass drug treatment programs be focused in areas with >1% prevalence of the infection, working under the assumption that areas with <1% prevalence are equivalent to areas of limited or no transmission. We carried out an additional assessment in low-prevalence areas and observed evidence of active transmission and clustering of antigen-positive persons. Our results imply that a 1% infection threshold may not be sufficient to capture all remaining reservoirs of transmission.

## Introduction

Lymphatic filariasis (LF) is one of 13 neglected tropical diseases (NTDs) known to chronically infect some of the worlds' poorest individuals [1]. While LF has been shown to be endemic in over 80 countries world-wide, it is one of six diseases that were deemed to be eradicable in 1993 by the International Task Force for Disease Eradication [2]. Since LF was made a priority by the World Health Organization (WHO) in 1997, there has been much progress in the control and elimination of LF across the globe [3]. In 2000, the WHO developed the Global Programme for the Elimination of Lymphatic Filariasis (GPELF) and established a goal to eliminate LF by 2020. A “two-pillar” approach has been implemented for the control and elimination of LF that focuses on the interruption of transmission through Mass Drug Administration (MDA) and limiting the disability caused by infection through morbidity management programs. The original definition of elimination used by the WHO was based on the demonstration that the microfilaria (Mf) or antigen prevalence at the community level was <1% and that the cumulative incidence in children born after the start of a MDA was less than 1 per 1000 children.

National mapping of lymphatic filariasis identifies areas requiring MDA by utilization of tests for microfilaremia or antigenemia. The immunochromatographic test (ICT), a rapid antigen test, is considered to be one of the most practical tools for the rapid mapping of endemic areas [4]. Mapping methods are generally based on convenience sampling to identify implementation units (administrative units, identified by the Ministries of Health, as the unit for implementing MDA) in need of MDA [5], [6]. These approaches to mapping facilitate programmatic decision-making, but because of the known heterogeneity of LF, microfoci of LF transmission may be missed when overall infection prevalence is low. Strategies for defining residual foci of transmission in low-prevalence settings are relevant to the global elimination effort, both from the perspective of targeting communities for MDA and for understanding surveillance requirements following cessation of MDA.

Haiti is one of only four LF-endemic countries in the Americas and bears 90% of the LF disease burden in the region [7]. The LF-causing filaria *Wuchereria bancrofti* has been historically documented in Haiti as far back as the 1700s, primarily transmitted by the *Culex quinquefasciatus* species [7]. Based on nation-wide mapping carried out in 2001, antigen prevalence ranged from zero to 45% among 6 to 11 year olds and 88% of the 120 communes that had been defined at the time had a prevalence greater than 1% [8]. In the current study, we conducted follow-up surveys to analyze potential transmission of LF in five communes that did not exceed this 1% threshold. Antigen surveys were performed in schools and selected antigen-positive children were defined as index cases for subsequent community surveys. Households near the residence of index cases were mapped and persons from a random sample of these households were tested for antigenemia using the ICT rapiddiagnostic. The analysis was designed to determine if active transmission of LF occurred in these settings and if infection prevalence exceeded the 1% threshold for MDA in some communities.

## Methods

### Ethics Statement

Surveys were conducted according to study protocols approved by the Centers for Disease Control and Prevention (CDC) and University of Notre Dame Institutional Review Boards (IRBs) and the Ethics Committee of Ste. Croix Hospital. During previous research in the area, researchers observed low rates of literacy in the population; therefore, all information regarding the study was translated into Haitian Creole, and verbal consent and assent were requested from parents and participants in both the school and community surveys. The consent form was read to potential study participants and/or parents. The reader of the consent form and a witness were then asked to sign the form to indicate the subject's agreement, in accordance with the IRB-approved protocol. Before surveys began in the schools, approval was obtained from the Haitian Ministry of Health and Population (MSPP), the Ministry of Education, department directors, and schoolmasters. Subsequently, meetings were held with parents to provide an opportunity to explain the survey and the potential risks and benefits to their child. Prior to the community survey, community leaders were informed of the survey's procedures and provided approval for the study to commence. At the time of the survey, household members were informed of survey objectives and procedures, at which time oral consent or assent was obtained for all participants.

### Low-Prevalence Study Sites

In 2001, national mapping for LF was performed, as previously described, using antigen testing of 100–250 schoolchildren aged 6–11 years of age per commune (a district-sized political entity in Haiti) across all Haitian communes [8]. Since financial resources were limited, communes of highest antigen prevalence (≥10% prevalence) were prioritized for MDA. Our evaluation focused on five, predominantly rural, communes in which antigen prevalence in the survey was ≤1%: Grand Goâve (0.8%), Hinche (1.0%), Thomazeau (0.6%), Moron (0.8%), and St. Louis du Sud (0.4%).

### School Surveys

Within each of the communes, five to seven schools were selected for antigen testing. These public and private schools were in urban and rural areas. Following the acquisition of informed consent and assent, as detailed above, blood samples were collected from students and tested as described below. ICTs were performed on all consenting children on site and additional blood was taken for ELISA testing upon return to laboratory facilities. Questionnaires were administered to a parent or guardian of ICT-positive children. The questionnaires were designed to identify autochthonous cases—defined as those children thought to have acquired the infection in the community where testing was conducted based on responses to questions about the absence of travel and residence in the same community for the past five years.

### Index Case Selection

Five to eight ICT-positive children were chosen from each commune as index cases for that area. Index cases were children identified as antigen-positive by ICT in the school survey, with recoded GPS coordinates who responded to the questionnaire, and, when possible, were chosen based on those with confirmatory ELISA results. The index cases became central points in the subsequent community surveys.

### Community Survey

For the community surveys, households of index cases defined the center for each testing radius. Circles of 50–75 meters were used in more densely populated urban or peri-urban areas, and circles of 100–250 meters were used in sparsely-populated rural settings. After index cases were identified, all consenting members of index households, and a systematic random sample of the neighboring households were selected for testing by ICT. All neighboring houses within the test radius were mapped using global positioning system (GPS) TerraSync (Sunnyvale, CA). In an effort to test 100 persons per community, approximately 20 households were chosen, based on an estimate of five persons per household (unpublished data). To select these 20 houses, the total number of houses in the zone was divided by 20 to determine the sampling interval. Houses were selected from a numbered list using a randomly selected starting point and this sampling interval. The questionnaire and methods used for blood collection and testing were the same as those used for the school survey. A total of 1,633 persons of all ages were evaluated in the community survey. For our study, subjects were included in the analysis if they had not been previously defined as an index case in the school study, received an ICT test result, and GPS coordinates were available for their households (n = 1290).

### Blood Collection and Serologic Testing

A blood specimen was collected from each person tested in the school or community survey. Filarial antigen-status was determined by ICT (Binax, Portland, ME) by trained laboratory personnel at the time of blood collection. Finger prick blood (100 µl) was collected and results were read at 10 minutes following application to the antigen test card. Technicians were supervised, and all efforts were made to uphold the quality of the test in regards to environment and timing. An additional 200 µl of blood was collected for confirmatory antigen testing. Collected blood was stored overnight at 4°C. The tubes were centrifuged the following day and the collected sera were stored in the field at 4°C for several days until return to Hôpital Ste. Croix where they were stored at −20°C. Sera were used for subsequent serologic assays, including confirmation of antigen status for persons with positive or questionable ICT results by Og4C3 antigen enzyme-linked immunosorbent-assay (ELISA, TropBio, Queensland, Australia). For the school survey and the subsequent community survey, ICT results were used as the indicator of antigen status for all persons; furthermore, autochthonous index cases were confirmed as antigen-positives by Og4C3 ELISA, the current gold standard for identifying circulating filarial antigens. Antigen-positive persons were treated with a single dose of diethylcarbamazine (DEC, 6 mg/kg). Due to the low percentage of ICT positive persons in each commune and the logistic difficulty of night blood surveys, microfilaria levels were not assessed.

### Data Analysis

Data were analyzed using SAS 9.3 (Cary, NC, USA), Epi Info 6 (CDC, Atlanta, USA) and ArcGIS (v. 9.3.1, Environmental Systems Research, Inc., Redlands, CA, USA). Univariate, Mantel Haenszel chi-square and logistic regression techniques were utilized.

The outcome of interest for this analysis was antigen positivity as denoted by the ICT results performed in the field. Two separate case definitions were employed as indicators of possible exposure in this analysis. The definition of index case only required a positive ICT among children tested in the school survey and the availability of GPS data. Index cases would therefore serve as potential, but unconfirmed, reservoirs of infection. A second, more stringent case definition was applied for children with positive Og4C3 results and who were defined as autochthonous cases based on their answers to the survey. These individuals were referred to as autochthonous index (AI) cases. The exposure of interest was the distance from each person tested to the nearest index or autochthonous index case. In order to determine the ordinal categories which best represent the distance to cases, a sensitivity analysis was performed for dichotomized distances of 10, 20, 40, 80 and 160 meters. Analysis of distance when using the AI case definition revealed no antigen-positive persons in the 59–99 m group, so the categories of 59–99 and 100+ meters were combined into a 60+ meter group, which was then used as the referent for the crude and multivariate regression analyses.

Potential confounders and effect modifiers, including age, gender and commune were also considered based on previous literature and anticipated heterogeneity among the communes. For the purpose of modeling, age was dichotomized into <15 years and ≥15 years, with the age of 15 or younger to denote school-aged children.

A spatial cluster analysis was performed on mapped households in the four communes recording antigen positivity. The analysis tested the spatial clustering of antigen-positive persons (excluding index cases) through the use of a Bernoulli model in SatScan (version 9.1.1, Boston, MA). A separate cluster analysis was performed for each of the four communes that included confirmed antigen-positives to better elucidate micro-clusters. Both general and isotonic simulations were performed on the commune-specific data, the latter of which accounts for the inverse relationship between risk and distance from the center of the cluster [9]. This type of simulation holds biological plausibility in representing the transmission patterns of vector-borne diseases.

## Results

**Figure 1:**
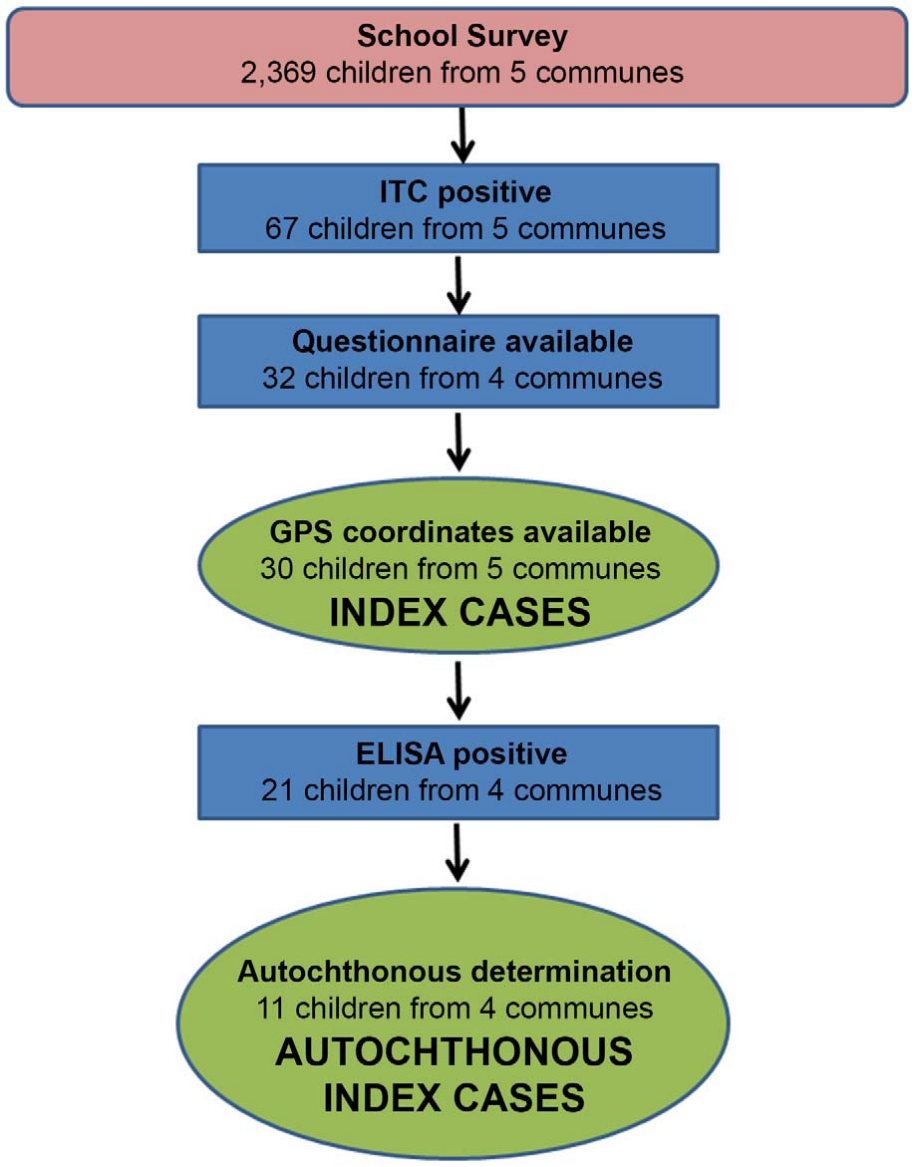
Index and autochthonous index case selection criteria from school survey data 2001–2003.

Of the 2,639 children tested (age range 4–17) in the school survey 67 (2.8%) were antigen-positive by ICT (Figure 1). Positive ICTs were observed for all five communities; however, following confirmatory ELISA testing, only 3 of the 5 communes presented higher antigen prevalence than previously observed in the original national survey. The school survey was used as guide for selecting the areas for the subsequent community survey. Each of the 67 ICT positives was followed up with confirmatory ELISA testing and was given a questionnaire to determine if their infection was autochthonous in origin. Using the aforementioned case definitions, 30 children were identified as index cases and 11 were identified as autochthonous index (AI) cases (see Figure 1 for derivation of case definition from source population).

**Figure 2:**
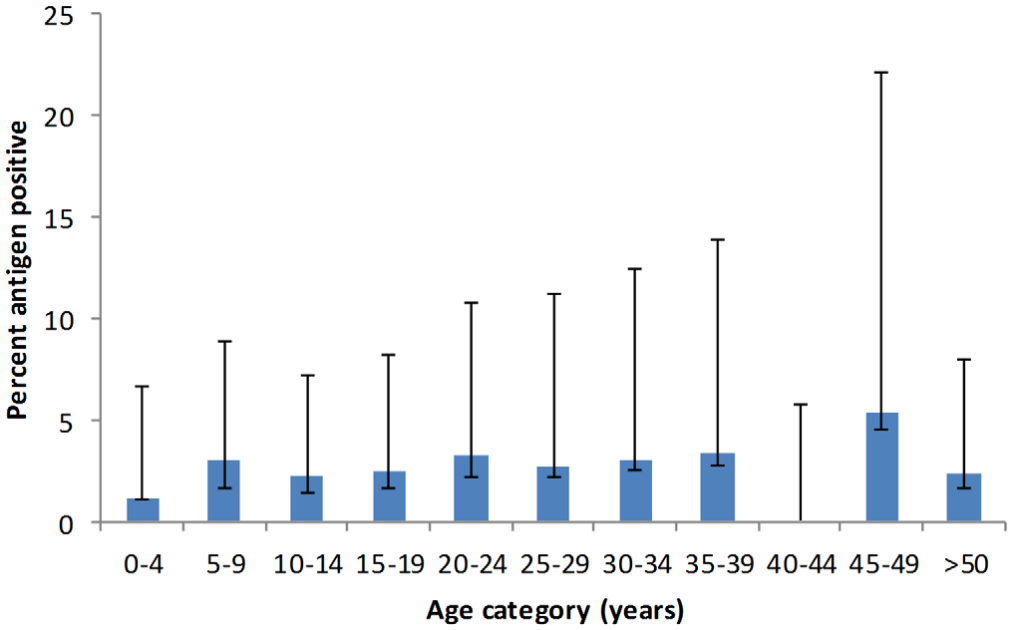
Prevalence of antigen positivity, identified by ICT status, by age category in the community survey (n = 1285).

While ICT results showed antigen positive cases from the original school survey in Moron, none of those results were confirmed by ELISA testing. Furthermore, the community survey showed no positive ICTs, thus all points within the Moron commune were excluded from the remaining analysis. Among the remaining communes, based on the results of the community survey, antigen prevalence was highest in Grand Goâve (4.35%), and lowest in St. Louis du Sud (0.82%). Females were slightly more represented in the tested population, but this difference was not statistically significant (Table 1). The community survey included a broad range of ages, from 2 to 90 years old (mean = 24). Overall, antigen prevalence in young children ages zero to four years was higher than expected (1.2%) for an area at low risk for transmission, antigen prevalence increased to 3.0% in older children (5 to 9 years old), after which point antigenemia was relatively stable for older age groups (Figure 2). Antigen prevalence in the community survey was related to commune and the distance from the index case (p = 0.0044), but not to gender or to living in an urban versus rural environment (Table 1).

**Table 1:** Characteristics of study population tested for filarial antigen status in the community survey*.

Variable	Total n	Antigen positive percent of total (n)	p-value**
**Commune**			***0.0136
Grand Goâve	299	4.35% (13)	
Hinche	276	3.99% (11)	
Moron	98	0.00% (0)	
St. Louis du Sud	244	0.82% (2)	
Thomazeau	373	1.88% (7)	
**Distance from index case (m)**			0.0044
<20	217	5.99% (13)	
20–59	413	1.94% (8)	
60–99	343	2.33% (8)	
100+	317	1.25% (4)	
**Age (years)**			0.7565
Age≥15	745	2.68% (20)	
Age<15	540	2.41% (13)	
**Gender**			0.7143
Male	545	2.75% (15)	
Female	742	2.43% (18)	
**Locale**			0.1693
Urban	202	3.96% (8)	
Rural	1088	2.30% (25)	

* Total n may differ depending on the number of persons responding to each variable.

** p-value was determined using a chi square analysis.

*** p-value was determined using Fisher's exact methods.

**Figure 3:**
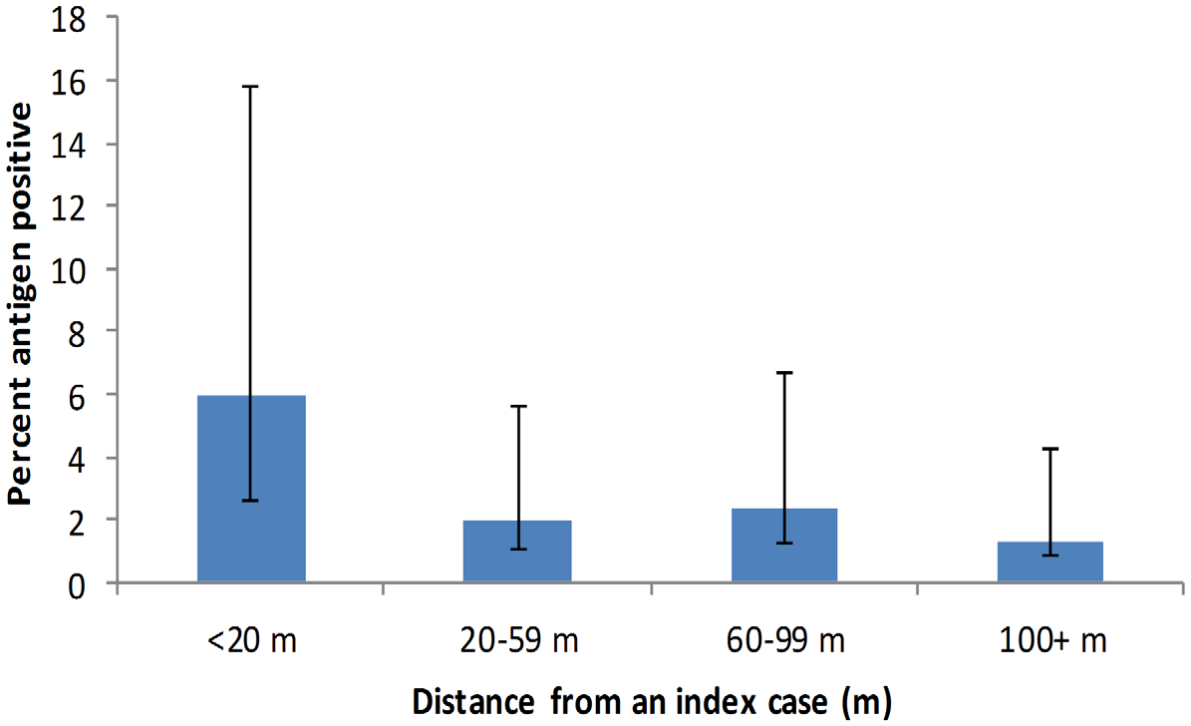
Antigen positivity by distance from an index case in the community survey (n = 1290).

A sensitivity analysis was conducted to evaluate the relationship of distance from an index case to antigen positivity. The relationship was strongest when a distance of 20 meters from the index case was employed (p = 0.0004), and was therefore used as the cutoff for the distance variable (Table 2). Antigen prevalence was highest for persons living within 20 meters of the index case, with decreasing antigen prevalence as distance from an index case increased (Figure 3). Furthermore, 40% of the persons testing antigen positive were found to live within 20 m of an index case.

**Table 2:** Crude sensitivity analysis for influence of distance from index case (m) on antigen status (n = 1290).

Classification	cPOR	95% CI	p-value*
<10	1.79	(0.73, 4.41)	**0.2554
≥10	1.00 (ref)		
<20	3.36	(1.64, 6.85)	0.0004
≥20	1.00 (ref)		
<40	2.39	(1.19, 4.78)	0.0113
≥40	1.00 (ref)		
<80	1.58	(0.73, 3.43)	0.2423
≥80	1.00 (ref)		
<160	3.07	(0.42, 22.68)	**0.3544
≥160	1.00 (ref)		

* p-value was determined using a chi square analysis.

** p-value was determined using Fisher's exact methods.

### Index Case Results

Crude odds ratios were calculated to evaluate the likelihood of being antigen-positive compared across the individual covariates: distance from index case, age, gender, locale (meaning urban or rural habitation) and commune. A distance of less than 20 m from an index case produced a crude prevalence odds ratio of 4.99 [95% CI 1.60, 15.51] when compared with distances of 100 m or more from an index case. All other individual covariates were evaluated for significance, but none besides distance of less than 20 m was statistically significant.

Multivariate logistic regression techniques were applied and evaluated for collinearity, interaction and confounding, and the final model is presented in Table 3 where the exposure of interest is distance from an index case. The odds of positive antigen status among persons living within 20 meters of an index case is 5.41 [95% CI 1.64, 17.83] times the odds of positive antigen status among persons living 100 meters or more from an index case, when controlling for age, gender and commune. The communes of Grand Goâve and Hinche showed significantly higher odds ratios for antigen prevalence (5.72 [95% CI 1.26, 25.90], and 7.17 [95% CI 1.53, 33.50] respectively) compared to Thomazeau.

**Table 3:** Multivariate model for the effect of distance from an index case on antigen status (n = 1187)*.

Variable	POR	95% CI
**Commune**		
Grand Goâve	5.72	**(1.26, 25.90)
Hinche	7.17	(1.53, 33.50)
St. Louis du Sud	3.16	(0.63, 15.78)
Thomazeau	1.00 (ref)	
**Distance from index case (m)**		
<20	5.41	**(1.64, 17.83)
20–59	1.45	(0.41, 5.13)
60–99	1.85	(0.54, 6.35)
100+	1.00 (ref)	
**Age (years)**		
Age>15	1.21	(0.58, 2.50)
Age<15	1.00 (ref)	
**Gender**		
Male	1.15	(0.56, 2.34)
Female	1.00 (ref)	

* Analysis controls for all other variable in the model. Moron was excluded from further analysis because it showed no positive results for antigen status.

** Denotes significant confidence interval.

### AI Results

The parallel analysis using the AI case definition determined that there were no AI cases in the initial school survey in Moron and St. Louis du Sud, thus both were excluded from further analysis. Similar results were obtained between the index and the AI case definitions in both the crude and multivariate analyses (Table 4). A proximity of less than 20 m to an AI case had a statistically significant increased odds of being antigen positive compared to distances of 60 meters or more from the AI case (cPOR 6.76 [95% CI 2.31, 19.78]) in the crude analysis, while no other covariates yielded statistically significant results. Multivariate logistic regression techniques identified an even larger increased odds of antigen positivity with close proximity to AI cases (6.70[95% CI 2.02, 22.21]) when controlling for age, gender and commune; and the communes of Grand Goâve and Hinche showed slightly higher odds of being ICT-positive when compared to Thomazeau; all of which were statistically significant.

**Table 4:** Multivariate model for the effect of distance from autochthonous index cases on antigen status (n = 797)*.

Variable	POR	95% CI
**Commune**		
Grand Goâve	1.30	(0.41, 4.09)
Hinche	2.14	(0.75, 6.16)
Thomazeau	1.00 (ref)	
**Distance from AI case (m)**		
<20	6.70	**(2.02, 22.21)
20–59	1.26	(0.44, 3.61)
60+	1.00 (ref)	
**Age (years)**		
Age>15	1.11	(0.49, 2.49)
Age<15	1.00 (ref)	
**Gender**		
Male	1.33	(0.60, 2.97)
Female	1.00 (ref)	

* Analysis controls for all other variables in the model. Moron and St. Louis du Sud were excluded from further analysis because no autochthonous index cases were identified in these communities.

** Denotes significant confidence interval.

### Spatial Cluster Analysis

**Figure 4:**
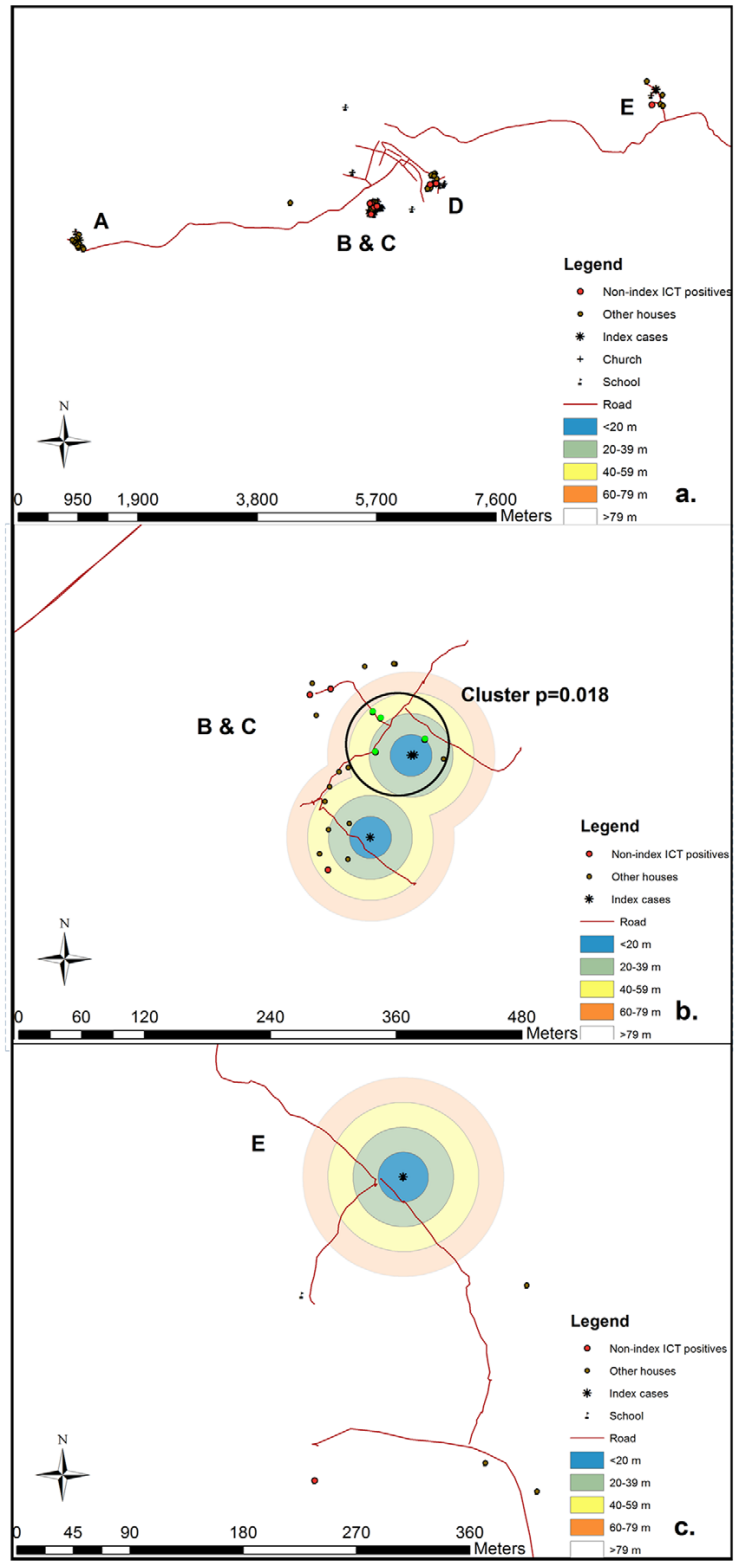
Maps of Hinche showing clusters of households around index cases. 4a gives an overall view of the households sampled within Hinche. Households containing index cases are denoted by a star and distinguished by letters A–E, households containing a non-index ICT positive person are denoted by a red dot, and households containing no ICT positive persons are denoted by a green dot. 4b shows an example of the proximity to index cases and clustering of households. Letters B and C denote the individual index cases, whose houses are represented by a star. Concentric rings at specified distances indicate which distance from index case category each household falls within. Households denoted by the bright green dot indicate houses that were identified as part of the cluster around index C. There was no significant clustering of households containing ICT positives for index B. 4c. shows the absence of clustered household containing ICT positive persons in proximity to index D.

Spatial analyses were carried out in each commune, looking at the clustering of antigen-positive persons compared to the total number of persons tested. The Bernoulli model analyzed spatial clustering of cases and non-cases from a total of 319 households, each with an average of four people tested per household. Results shown in Table 5 demonstrate statistically significant clustering of cases in Hinche and Thomazeau, when evaluated at the 5% significance level in both the general and isotonic Bernoulli analyses. Examples of clustering can be seen in Figure 4.

**Table 5:** Clustering of antigen positive households by commune using SatScan, version 9.1.1*.

*General Bernoulli cluster analysis*					
	Most likely cluster			Total significant clusters	
Commune	p-value **	Radius (m)	Number of cases	Number	Range of radii (m)
Grand Goâve	0.137	10	3	0	NA
Hinche	0.0025	0	3	4	0–370
St. Louis du Sud	0.53	3840	2	0	NA
Thomazeau	<0.0001	0	12	5	0–2,310

*Isotonic Bernoulli cluster analysis* ***					
	Most likely cluster			Total significant clusters	
Commune	p-value **	Radii for steps 1–3 (m)	Number of cases	Number	Range of radii (m)
Grand Goâve	0.191	10	3	0	NA
Hinche	0.0056	0, 120, 1,010	10	2	0–1,010
St. Louis du Sud	0.238	700, 7,000, 7,020	2	0	NA
Thomazeau	<0.0001	0, 70, 5,830	16	2	0–5,830

* Moron was excluded from further analysis because it showed no positive results for antigen status.

** p-value was determined using a chi square analysis.

*** The isotonic Bernoulli cluster analysis decreases the effect as distance from the center increases. This analysis is carried out in 3 steps with increasing radii.

## Discussion

### School Survey

The original mapping for Haiti, carried out in 2001, identified the communes of Grand Goâve, Hinche, Moron, St. Louis du Sud and Thomazeau as areas of low antigen prevalence (≤1%). As transmission of lymphatic filariasis was presumed to not be occurring, the original conclusion was that MDA was not required in these areas. A subsequent school survey was conducted to determine if there was evidence of ongoing transmission in such areas. The results from our survey showed both higher than expected (>1%) antigen prevalence in three of the five communes and unexpected presence of autochthonous cases (Figure 1). These observations provide evidence that transmission of LF is occurring in settings previously identified as below the prevalence threshold that would trigger a MDA. We do not know whether the results we have observed in low-prevalence areas reflect historic foci not detected through previous surveys, or recently established transmission as a consequence of population migration, or expansion of vector populations [10]. These results were shared with the MSPP and led to the decision to carry out MDA across all Haitian communes, independent of the initial mapping results.

The statistically significant clustering of antigen-positive cases and increased odds of antigen positivity that were observed in the crude and multivariate analyses, as a function of distance to index and AI cases, all suggest that transmission might be occurring in microfoci, that is, among people living in very close proximity to one another, posing challenges for current mapping strategies. The model demonstrated a statistically significant increased likelihood of having a positive ICT result when residing within 20 meters of an index or AI case controlling for age, gender and commune, suggesting that antigen-positive children can serve as indicators of microfoci of transmission, and that proximity to these microfoci may be associated with the risk of acquiring LF.

Risk associated with proximity to infected persons becomes of particular interest as communities see fewer and fewer instances of new infections. Different studies have reached independent conclusions regarding the possible risk. One study from Brazil found that one antigen-positive individual did not seem to pose a significant risk for transmission, as no one in the vicinity had become infected in the 10 years he lived in this non-endemic community [11]. However, this study was only observational and based on a single individual and therefore may not be generalizable to the overall population.

Washington et al. addressed the probability of acquiring LF as a function of the distance from antigen-positive cases through an analysis of changes in antifilarial antibodies [12]. This study determined that for every 10 meter increase in distance from an antigen-positive case, there was a 5.6% decrease in IgG1 antibody levels, when controlling for age, gender and treatment status (p = 0.04) [12]. These observations coupled with our present study indicate substantial risk with spatial proximity to an antigen-positive person in both exposure as well as acquisition of LF arguing that clustering may play a substantial role in transmission dynamics.

The latest published research pertaining to the clustering and identification of “hotspots” of LF was performed by Joseph et al [13], in which researchers examined the spatial clustering of LF in Samoa. This study looked at the clustering not only antigen positive, but also microfilaremic and antibody positive persons. Their results revealed statistically significant clustering of antigen positive individuals in three of the tested communities with radii ranging from 0 to 1160 meters. Our study complements this work by documenting the presence of clustering prior to the implementation of MDA in low prevalence settings.

The final multivariate logistic regression model included commune as a significant variable, consistent with the conclusion that differences in transmission exist between communes (Table 3). Differences in the transmission of LF are likely due to different physical environments or population densities, either of vectors or humans, which may be more compatible with transmission of LF and could warrant further exploration. We did not collect data on mosquito densities for this study; however, it is likely that clustering and dispersal of infections are influenced by the behavior and flight range of the vector mosquito as well. Future studies also should examine the micro-environment surrounding the households of study participants to better address possible heterogeneity.

### Limitations

This study is a preliminary analysis of the factors associated with antigen prevalence in five communes in Haiti in 2003, and the findings may not be generalizable to all endemic settings. Infection risk could not be established definitively in this cross-sectional study, and we suggest a cohort study be conducted in order to confirm these results. The cross-sectional study design also does not allow for chronology to be established so there is no way to determine if cases identified in the school survey were infected before or after their ICT-positive neighbors. Thus, although we can argue that antigen-positive children were indicators of community infection, we could not determine the actual reservoir or source of this transmission. Due to the low percentage of ICT positive persons in each commune and the logistic difficulty of night blood surveys, microfilaria levels were not assessed. While generally highly specific, the ICT is not considered the most accurate test for LF infection because of problems with test interpretation in the field [14]. Testing using Og4C3 ELISA provides a quantitative measure of circulating antigens and is generally accepted as being a more sensitive test of antigenemia; however, due to financial and logistical considerations, the ICT was used for all participants in the study.

Presence of microfilaria as well as biting rates from the vector would allow for the calculation of rate of transmission, and transmission risk; however, such tests were not performed for this study. We used antigen positivity as an indicator of transmission in lieu of the acquisition of such entomologic data which can be challenging and expensive to collect, especially in the context of short term surveys.

Since this study was carried out in low-prevalence settings, there were few persons found to be antigen-positive. This is a challenge of sampling in a low-prevalence setting and it is accompanied by decreased statistical power. Lastly, in order to evaluate the exposure of interest, we required that GPS coordinates be available, in addition to ICT results. Since this information was not available for all study participants, our sample size was reduced.

### Conclusions

We have demonstrated that transmission, using antigen prevalence as a proxy, is occurring in areas that had previously been categorized as areas with low risk of LF transmission, suggesting that areas of low-prevalence may not be without transmission risk. The nation-wide mapping techniques in 2001 revealed a prevalence of ≤1% antigenemia for all five communes in our study, but we observed prevalence values ranging from zero to 5.6% in the school survey and 0 to 4.35% in the community survey within those same 5 communes.

The identification of autochthonous index cases indicates that transmission is occurring at the level of microfoci. Since our analysis revealed that living within 20 meters of an index case significantly increased the likelihood of being antigen-positive, such microfoci may represent a particular challenge in terms of surveillance following MDA. MDA is expected to reduce infection prevalence to the point that only small isolated foci of transmission are expected to remain. The concern that these foci might represent groups of persons who are systematically noncompliant has been hypothesized on several occasions [15], [16], [17]. How long such microfoci can persist is unknown. In any case, the presence of such foci should be addressed and the school survey strategy we have described may represent one approach to detecting such foci through active surveillance, as proposed by Huppatz et al. [18]. Such efforts will be aided by the development of new and more sensitive diagnostic tools, based on the detection of parasite-specific antibody [18], [19].

Ramaiah et al. reported residual microfilaria prevalence ranging from 0.03 to 0.43% in the population when tested annually over a period of 20 years post MDA [20]. It is impractical to require a surveillance period of 20 years post MDAs; however, increasing the period of surveillance from five years to ten years, prior to certification, might be necessary to ensure that transmission has indeed stopped or at least slowed to a point which cannot sustain the filarial lifecycle. Additional research is needed to address this issue. With a comprehensive program and stringent monitoring and evaluation of remaining infection we can make greater strides towards the elimination of lymphatic filariasis.
